# Chrysomycin A Inhibits the Proliferation, Migration and Invasion of U251 and U87-MG Glioblastoma Cells to Exert Its Anti-Cancer Effects

**DOI:** 10.3390/molecules27196148

**Published:** 2022-09-20

**Authors:** Dong-Ni Liu, Man Liu, Shan-Shan Zhang, Yu-Fu Shang, Fu-Hang Song, Hua-Wei Zhang, Guan-Hua Du, Yue-Hua Wang

**Affiliations:** 1 Beijing Key Laboratory of Drug Target Identification and New Drug Screening, Institute of Materia Medica, Chinese Academy of Medical Sciences & Peking Union Medical College, Beijing 100050, China; 2 School of Light Industry, Beijing Technology and Business University, Beijing 100048, China; 3 School of Pharmaceutical Sciences, Zhejiang University of Technology, Hangzhou 310014, China

**Keywords:** glioblastoma, chrysomycin A, U251 glioblastoma cell, U87-MG glioblastoma cell

## Abstract

Chrysomycin A (Chr-A), an antibiotic from Streptomyces, is reported to have anti-tumor and anti-tuberculous activities, but its anti-glioblastoma activity and possible mechanism are not clear. Therefore, the current study was to investigate the mechanism of Chr-A against glioblastoma using U251 and U87-MG human cells. CCK8 assays, EdU-DNA synthesis assays and LDH assays were carried out to detect cell viability, proliferation and cytotoxicity of U251 and U87-MG cells, respectively. Transwell assays were performed to detect the invasion and migration abilities of glioblastoma cells. Western blot was used to validate the potential proteins. Chr-A treatment significantly inhibited the growth of glioblastoma cells and weakened the ability of cell migration and invasion by down regulating the expression of slug, MMP2 and MMP9. Furthermore, Chr-A also down regulated Akt, p-Akt, GSK-3β, p-GSK-3β and their downstream proteins, such as β-catenin and c-Myc in human glioblastoma cells. In conclusion, Chr-A may inhibit the proliferation, migration and invasion of glioblastoma cells through the Akt/GSK-3β/β-catenin signaling pathway.

## 1. Introduction

Glioblastoma (GBM) is the most frequent and fatal intracranial tumor characterized by rapid proliferation, migration, invasion and massive angiogenesis [1,2]. Furthermore, GBM usually encroaches on the circumambient normal brain tissue resulting in an obscure boundary between the tumor and the normal part due to its high degree of heterogeneity and aggressiveness. Therefore, the tumor tissue cannot be resected thoroughly by surgical intervention; subsequently, adopting radiotherapy and chemotherapy could prolong the duration of survival for GBM patients to some extent [3]. Temozolomide was accepted as the standard first-line chemotherapy but mostly followed with recurrence in GBM patients within 6 months [4]. Hence, developing original medicable chemotherapy drugs for glioblastoma is of great importance.

**Figure 1:**
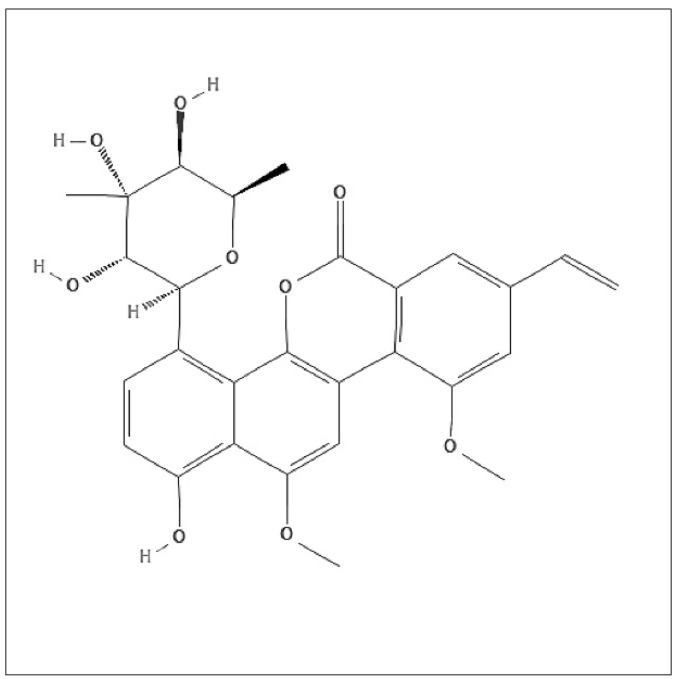
Structure of chrysomycin A.

In 1955, chrysomycin was first discovered from an unknown Streptomyces described as slender greenish-yellow needles or crystallized rods [5]. Chrysomycin A (Chr-A) (Figure 1, C28H28O9 and MW508.52), the major component, differs from chrysomycin B, the second component with the vinyl group of the 8-position [5], thus showing cytotoxicity towards cancer cells [6,7]. These years, conditions and preparation of Chr-A production has been greatly optimized, which is of great significance to its new drug development process [8,9]. Studies have been done to prove antibiotic activity, anti-tumor activity, anti-tuberculosis activity and anti-neuroinflammation activity of Chr-A [10,11,12,13]. However, the potential ability of Chr-A against glioblastoma is rarely reported.

Epithelial-mesenchymal transition (EMT), responsible for mediating invasion and migration, contributes a lot to the progression of multiple cancers through facilitating initiation and aggression of GBM [14,15]. N-cadherin, Snail, slug and matrix metalloproteases (MMP2 and MMP9) are essential EMT markers which are reported to be at high expression levels to enable cancer cells to become more motile and invasive [16]. PI3K/Akt/mTOR signaling pathway and Wnt/β-catenin signaling pathway are two essential regulation pathways for glioblastoma, found to be activated excessively under pathological conditions functioning to mediate cell proliferation, EMT process, apoptosis, autophagy, metabolism and angiogenesis [17]. Akt is a key point in a network affecting the progress of GBM, which could not only stimulate cell survival by activating mTOR, but could facilitate metastasis through interacting with Wnt signaling [18,19]. PI3K-activated Akt could inactivate GSK-3β with its phosphorylation [20]. Following GSK-3β in Wnt signaling, β-catenin then gets involved in the EMT process by binding with cadherin or mediating MMPs [21] and entering into the nucleus to promote Wnt-target genes, such as c-Myc, cyclin D1 [22].

Given these findings, we investigated whether Chr-A could suppress cell proliferation, migration, invasion of U251 and U87-MG cells and the possible molecular mechanisms.

## 2. Results

### 2.1. Chr-A Inhibits Viability and Proliferation of U251 and U87-MG Cells

**Figure 2:**
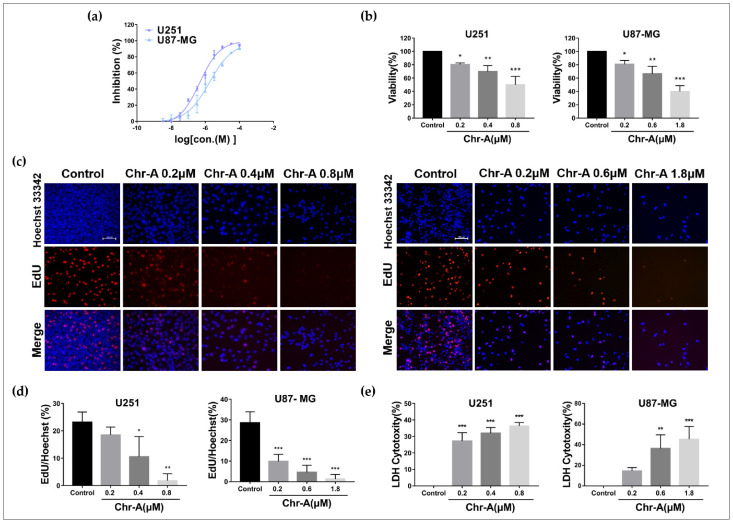
Chr-A causes proliferation inhibition and cytotoxicity rise in the U251 and U87-MG cells. (**a**) The CCK8 assay shows that Chr-A inhibited multiplication of the U251 and U87-MG cells with IC50 at 0.475 μM and 1.77 μM, respectively, after treatment for 48 h. (**b**) The CCK8 assay shows that Chr-A inhibited the viability of the U251 and U87-MG cells. (**c**,**d**) EdU-synthesis detection shows that Chr-A inhibited DNA synthesis in the U251 and U87-MG cells. Red fluorescence represents EdU positive cells, blue fluorescence represents Hoechest 33342 positive cells. Scale bar = 100 μm. (**e**) LDH detection shows that Chr-A increased cytotoxicity of the U251 and U87-MG cells. Data are demonstrated as mean ± SD (*n* = 3), * *p* < 0.05, ** *p* < 0.01 and *** *p* < 0.001 vs. the control group.

To figure out the effect of Chr-A on viability and proliferation of human glioblastoma, we adopted a CCK8 assay first, and our results showed that Chr-A treatment for 48 h inhibited the growth of the U251 and U87-MG cells at different doses (Figure 2a), and the IC50 values were 0.475 μM and 1.77 μM, respectively. Subsequently, we selected 0.2, 0.4 and 0.8 μM of Chr-A in the U251 cells and 0.2, 0.6 and 1.8 μM of Chr-A in the U87-MG cells to investigate the anti-glioblastoma efficacy of Chr-A and the possible mechanisms. Meanwhile, the viability of the U251 and U87-MG cells was significantly inhibited by Chr-A at different concentrations (Figure 2b). Furthermore, EdU-DNA synthesis ability detection was performed to verify the influence of Chr-A on the proliferation of human glioma cells, and the results showed that Chr-A treatment notably decreased EdU-positive cells with inhibiting the U251 and U87-MG cells proliferation (Figure 2c,d). In addition, Chr-A promoted LDH release which represents the membranous disruption out of apoptosis or death of the U251 and U87-MG cells (Figure 2e). Together, Chr-A showed an inhibitory role for viability and multiplication in U251 and U87-MG cells.

### 2.2. Chr-A Attenuates Migration and Invasion of U251 and U87-MG Cells

**Figure 3:**
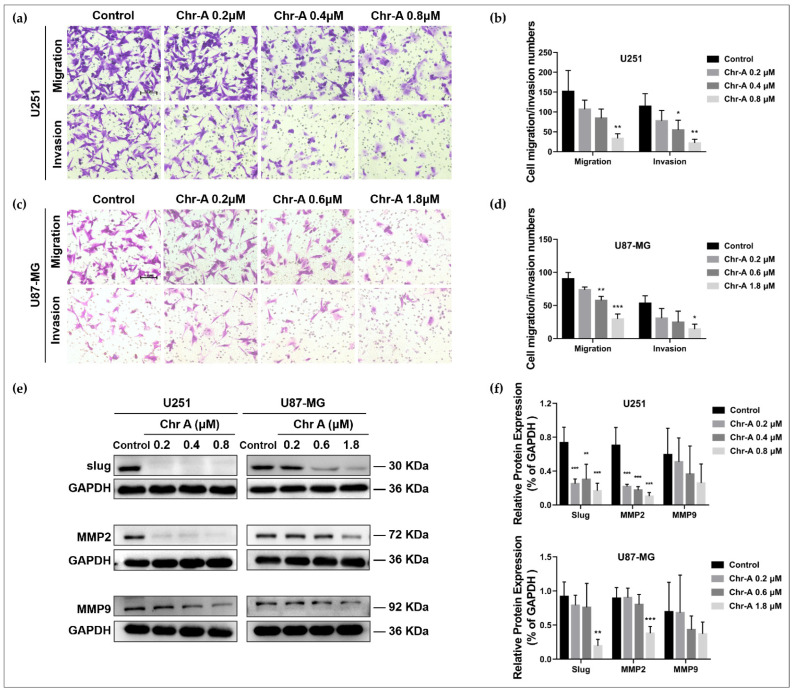
Chr-A suppresses migration and invasion of the U251 and U87-MG cells. (**a**,**b**) The Transwell assay shows that Chr-A restrained metastasis and invasiveness of the U251 cells (*n* = 3). Scale bar = 100 μm. (**c**,**d**) The Transwell assay shows that Chr-A restrained metastasis and invasiveness of U87-MG cells (*n* = 3). Scale bar = 100 μm. (**e**,**f**) The Western blot results show that Chr-A downregulates the expression of slug, MMP2 and MMP9 (*n* = 4). Data are demonstrated as mean ± SD, * *p* < 0.05, ** *p* < 0.01 and *** *p* < 0.001 vs. the control group.

Migration and invasion abilities are of great significance to the progression of glioblastoma. Our results from the Transwell assay showed that the number of cells that migrated or invaded were remarkably lessened in the U251 and U87-MG cells after Chr-A treatment at different concentrations (Figure 3a–d), demonstrating that metastasis and invasiveness of glioblastoma cells were restrained in the presence of Chr-A. Furthermore, Western blot verified the expression of pro-invasion factor, slug, and the metastasis related proteins, MMP2 and MMP9. Compared with the control group, Chr-A significantly downregulated the expression of slug and MMP2 in the U251 and U87-MG cells, while Chr-A downregulated MMP9 with barely a noticeable difference (Figure 3e,f). These results showed that Chr-A suppressed migratory aptitude and invasiveness of glioblastoma cells.

### 2.3. Chr-A Mediated Akt/GSK-3β/β-Catenin Signaling Pathway in U251 and U87-MG Cells

**Figure 4:**
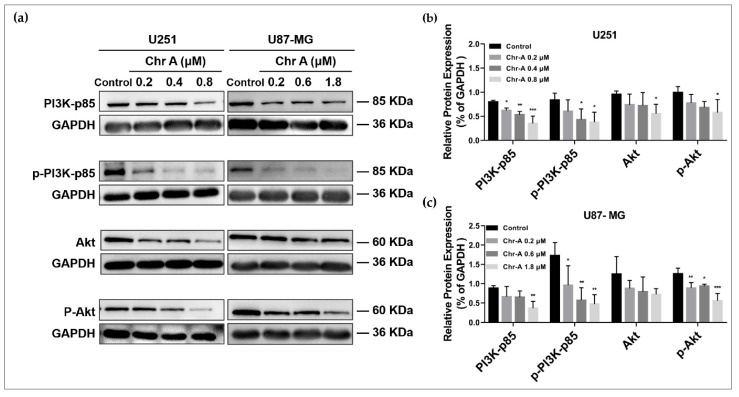
Chr-A regulates PI3K/Akt pathway in the U251 and U87-MG cells. (**a**) Western blot shows that Chr-A downregulated PI3K-p85, Akt, p-PI3K-p85 and p-Akt in the U251 and U87-MG cells. (**b**) Relative protein expression of PI3K-p85, Akt and their phosphorylated forms in the U251 cells. (**c**) Relative protein expression of PI3K-p85, Akt and their phosphorylated forms in U87-MG cells. Data are demonstrated as mean ± SD (*n* = 4), * *p* < 0.05, ** *p* < 0.01 and *** *p* < 0.001 vs. the control group.

**Figure 5:**
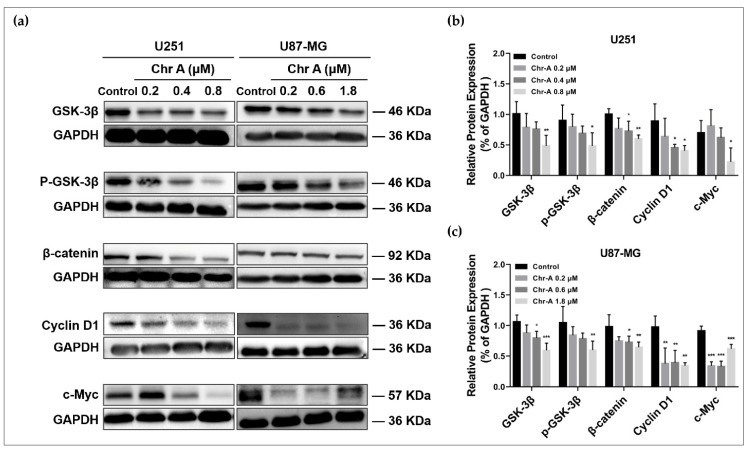
Chr-A mediates the Wnt/β-catenin pathway in the U251 and U87-MG cells. (**a**) Western blot shows that Chr-A downregulates protein expression levels of GSK-3β, p-GSK-3β, β-catenin, c-Myc and cyclin D1 in the U251 and U87-MG cells. (**b**) Relative protein expression of GSK-3β, p-GSK-3β, β-catenin, c-Myc and cyclin D1 in the U251 cells. (**c**) Relative protein expression of GSK-3β, p-GSK-3β, β-catenin, c-Myc and cyclin D1 in the U87-MG cells. Data are demonstrated as mean ± SD (*n* = 4), * *p* < 0.05, ** *p* < 0.01 and *** *p* < 0.001 vs. the control group.

To investigate the potential mechanisms on which Chr-A may exert anti-glioblastoma activity in human glioblastoma cells, the western blot analysis was conducted. The PI3K/Akt/mTOR signaling pathway and Wnt/β-catenin signaling pathway were usually excessively activated to regulate multiple biological process of glioblastoma including cell proliferation and metastasis. PI3K-activated Akt could inactivate GSK-3β followed by multiple downstream effects; thus, we proposed the hypothesis that Chr-A may inhibit glioblastoma through the Akt/GSK-3β/β-catenin pathway. The results showed that the protein expression of PI3K-p85, p-PI3K-p85, Akt and p-Akt were significantly decreased with the increase in concentrations of Chr-A in the U251 cells, suggesting that Chr-A may downregulate Akt with the downregulation of PI3K (Figure 4a,b). Furthermore, in the U87-MG cells, the protein expression of PI3K-p85, p-PI3K-p85 and p-Akt were significantly decreased with the increase in concentrations of Chr-A (Figure 4a,c). Taken together, the results suggest that Chr-A works against glioblastoma by downregulating PI3K-mediated Akt. Meanwhile, downregulation of GSK-3β, p-GSK-3β, β-catenin and their downstream, c-Myc and cyclin D1 using the Chr-A treatment indicates that Chr-A may exert anti-glioblastoma activity by downregulating the Akt/GSK-3β/β-catenin pathway (Figure 5). Interestingly, there was an increase for the expression of c-Myc in the U251 cells with no significance (Figure 5b).

## 3. Discussion

Glioblastoma is a common tumor of malignancy in clinical practice, which accounts for about 57% of malignant tumors of the central nervous system. Although great progress in medical technology has been made in recent years, the prognosis of glioblastoma is still not ideal [4]. The five-year survival rate for glioblastoma patients is still lower than 10% [4,23]. Temozolomide (TMZ) has been widely accepted as a typical first-line chemotherapy for glioblastoma, improving the prognosis positively but including nonnegligible adverse effects [24]. Thus, there is an urgent need to develop effective therapeutics for glioblastoma.

In the current study, we investigated and verified the anti-glioblastoma activity and possible mechanisms of Chr-A in U251 and U87-MG cells. Our research showed that Chr-A effectively inhibited viability and proliferation of U251 and U87-MG cells, reduced EdU synthesis and augmented LDH release, as well as attenuated migration and invasion through downregulation of slug and MMP2 in U251 and U87-MG cells. Furthermore, decreased expression of Akt-affected GSK-3β and their downstream proteins including β-catenin, c-Myc and cyclin D1 in U251 and U87-MG cells after Chr-A treatment for 48 h implied that Chr-A may exert an anti-glioblastoma action via the Akt/GSK-3β/β-catenin signaling pathway in vitro.

The EMT process facilitates the transition from epithelial neoplasms to metastatic tumors, which may be of significance for glioblastoma progression and chemotherapy resistance [25,26]. Mesenchymal markers, including N-cadherin, slug, snail and metastasis-related genes including MMP2, MMP9 and TIMP3 were widely investigated and verified in glioma remedy mechanisms [27,28]. Herein, the reduced expression of MMP2 and slug evidently corresponded to the effect that Chr-A suppresses metastasis and invasiveness in U251 and U87-MG cells with various concentrations of treatment for 48 h as the Transwell assay showed.

The PI3K/Akt/mTOR signaling pathway and Wnt/β-catenin signaling pathway become abnormally activated to regulate cytoskeletal rearrangement, metabolism, apoptosis and angiogenesis in GBM [29,30,31]. The two pathways function to weaken glioblastoma complementarily. Phosphoinositide 3-kinase (PI3K) is equipped with p85 regulatory subunits and p110 catalytic subunits. It is reported that p85 regulatory subunits promote the activation of p110 catalytic subunits leading to the formation of PIP3 with phosphorylation of PIP2 [32]. Subsequently, Akt recognizes the accumulation of PIP3 and targets downstream to mediate cell growth, cell cycle progression and apoptosis [33]. Moreover, the activation of Akt by PI3K can inactivate GSK-3β with its phosphorylation at the Ser9 site [20], thus affecting β-catenin phosphorylation at the N-terminus followed by β-catenin assembling and its’ nuclear translocation, which interacts with T-cell factor/lymphoid enhancing factor (TCF/LEF) to influence the downstream involving EMT related genes and proliferation regulating genes, such as c-Myc, cyclin D1, slug, MMP2 and MMP9 [19,33,34,35,36]. Herein, we observed a significant decrease expression level of total PI3K-p85, p-PI3K-p85, total Akt, p-Akt, GSK-3β and p-GSK-3β, as well as their downstream, β-catenin, c-Myc and cyclin D1 after treatment with Chr-A at different concentrations for 48 h in U251 and U87-MG cells, suggesting that Chr-A could downregulate PI3K-activated Akt followed by downregulation of GSK-3β, thus attenuating expression of β-catenin and downstream, c-Myc, cyclin D1, slug, MMP2 and MMP9 to inhibit proliferation and the EMT process of human glioblastoma cells.

## 4. Materials and Methods

### 4.1. Reagents

Chrysomycin A was provided by Prof. Hua-Wei Zhang (Zhejiang University of Technology, Hangzhou, China) and Prof. Fu-Hang Song (Beijing Technology and Business University, Beijing, China). DMEM and FBS (Cat# 164210-50) were obtained from Gibco BRL (Grand Island, NY, USA) and Procell (Wuhan, China), respectively. The EdU Apollo^®^ 567 In Vitro Imaging Kit was obtained from RiboBio (Guangzhou, China). The LDH Assay Kit was obtained from Dojindo (Tokyo, Japan). The anti-Slug antibody (Cat# 9585), anti-MMP2 antibody (Cat# 87809), anti-MMP9 antibody (Cat# 13667), anti-PI3K-P85 antibody (Cat# 4257), anti-p-PI3K-P85 antibody (Cat# 4228), anti-Akt antibody (Cat# 9272), anti-p-Akt antibody (Cat# 9271), anti-GSK-3β antibody (Cat# 9315), anti-p-GSK-3β antibody (Cat# 5558), anti-β-catenin antibody (Cat# 8480), anti-c-Myc antibody (Cat# 13987), anti-Cyclin D1 antibody (Cat# 2978) and anti-GAPDH antibody (Cat# 5174) were obtained from CST (Beverley, CA, USA).

### 4.2. Cell Culture and Cell Viability Assay

The cell bank of the Chinese Academy of Sciences (Beijing, China) offered the U251 cells and U87-MG cells. The cells were cultured in DMEM containing 10% FBS in an incubator at 37 °C with a humidified atmosphere of 5% CO_2_. The cells were seeded into a 96-well plate at the density of 5 × 10^3^ cells in each well. Chr-A (100 mM, diluted in DMSO) was further diluted in DMEM before adding to the cells after 24 h of adherent growth. After Chr-A treatment for 48 h, each well was processed with a CCK-8 solution followed by incubation for 1 h in a 37 °C incubator, and then the absorbance at 450 nm was determined by a microplate reader (MD, Sunnyvale, CA, USA). The experiments were performed in triplicate.

### 4.3. EdU-DNA Synthesis Assay

The DNA synthesis activity was determined by an EdU Apollo^®^ 567 In Vitro Imaging Kit. The cells were seeded into 96-well plates at a density of 1 × 10^4^ in each well for 24 h of adherent growth. Then, the culture media was replaced to serum-free DMEM with Chr-A at different concentrations. In addition, 0, 0.2, 0.4 and 0.8 μM Chr-A were added to the U251 cells; 0, 0.2, 0.6 and 1.8 μM Chr-A were added to the U87-MG cells for 48 h. After the Chr-A treatment, 50 μM of EdU was added to each well and cultured for another 3 h at 37 °C and went through a fixation in 4% paraformaldehyde for 30 min followed by permeation in 0.5% Triton-X 100. An amount of 10 μM of Apollo 567 was then used to stain the cells, and after 30 min of staining, the cells were then further stained by Hoechst 33,342 for 30 min and photographed using a fluorescence microscope (Nikon, Tokyo, Japan). The experiments were performed in triplicate.

### 4.4. Lactate Dehydrogenase (LDH) Detection

The LDH Assay Kit was adopted to measure LDH release of human glioblastoma cells. The Chr-A. 1 × 10^5^ cells were seeded in each well of a 96-well plate with DMEM containing 5% FBS firstly for 24 h of adherent growth. Then 0, 0.2, 0.4 and 0.8 μM Chr-A were added to the U251 cells; 0, 0.2, 0.6 and 1.8 μM Chr-A were added to the U87-MG cells for 48 h. After the Chr-A intervention, each well was appended with 100 μL of working solution for 30 min followed by a stop solution; the absorbance at 490 nm was measured by a SpectraMax M5 spectrophotometer (Molecular Devices, Sunnyvale, CA, USA). The experiments were performed in triplicate.

### 4.5. Transwell Migration and Invasion Detection

Cell metastasis and invasiveness abilities of human glioblastoma were detected by a Transwell assay. One hundred and fifty microliter cell suspension containing 1 × 10^5^ cells in DMEM with 1% FBS were added into the upper chamber of the 24-well Transwell chambers (8 μM pores, Corning costar, USA) at 37 °C for 3 h of adherent growth. Subsequently, the culture medium in the chamber was replaced with serum-free DMEM containing Chr-A at 0, 0.2, 0.4 and 0.8 μM in the U251 cells and 0, 0.2, 0.6 and 1.8 μM in the U87-MG cells. Meanwhile, 800 μL of DMEM containing 10% FBS was added into the lower chamber. After incubation at 37 °C for 24 h, 4% paraformaldehyde (Solarbio, Beijing, China) was used to fix the migrated or invaded cells for 30 min followed by staining with 1% crystal violet (Solarbio, Beijing, China) for 15 min. Then the cells were photographed (Nikon, Tokyo, Japan) in three independents per well. The Transwell assay for invasion was similar to the assay for migration, but Matrigel (diluted with DMEM at a ratio of 1:8), which has to be pre-coated in the Transwell upper chamber before adding the cell suspension, was indispensable. The cells that migrated or invaded were photographed with fluorescence microscope (Nikon, Tokyo, Japan), the image was processed by Image J and the difference was counted with GraphPad prism7 software. The experiments were performed in triplicate.

### 4.6. Western Blot Analysis

Proteins from the U251 and U87-MG cells after Chr-A treatment for 48 h were extracted with a RIPA lysis buffer (ApplyGen, Beijing, China) for 30 min in an ice water bath. To facilitate the extraction, centrifugation at 12,000 rpm was performed for 15 min at 4 °C. The supernatant fluid was obtained and quantified by a BCA Protein Assay Kit (Applygen, Beijing, China). The proteins at 30–40 μg were added to each lane and separated with 10% SDS-PAGE gel at a constant 120 V followed by transformation to PVDF membranes (Millipore, Billerica, MA, USA) at constant 200 mA. Then, the membranes were blocked with 5% skimmed milk in a TBST buffer for 2 h and incubated with corresponding primary antibodies at 4 °C overnight. After that, the membranes were washed 4 times with the TBST followed by incubation with a secondary antibody conjugated with horseradish peroxidase for 2 h at room temperature. Next, an ECL hypersensitive luminescence solution was used to visualize the membranes with proteins. The grayscale value of the bands on the images was analyzed by Image J software. The experiments were performed in quadruplicate.

### 4.7. Statistical Analysis

The measurement data were analyzed using the GraphPad prism7 software, and they were demonstrated as mean ± SD. Ordinary one-way ANOVA multiple comparisons were applied to determine the differences between the various study groups. *p* < 0.05 was considered statistically significant.

## 5. Conclusions

In conclusion, we first gave evidence that Chrysomycin A effectively inhibited proliferation, viability, migration, invasion and induced cytotoxicity of U251 and U87-MG cells. Furthermore, we revealed that Chr-A could exert anti-glioblastoma activity through the Akt/GSK-3β/β-catenin pathway.

## Data Availability

Not applicable.

## Sample Availability

Samples of the compounds are not available from the authors.
